# MiR-371-5p regulates trophoblast cell proliferation, migration, and invasion by directly targeting ZNF516

**DOI:** 10.18632/aging.205826

**Published:** 2024-05-17

**Authors:** Zhi Qiu Xie, De Fang Chen, Jie He, Linsheng Zhong, Guanzheng Luo, Ming Fang

**Affiliations:** 1 Electrocardiogram Room, Guangdong Women and Children Hospital, Guangzhou 511442, China; 2 The Affiliated Guangdong Second Provincial General Hospital of Jinan University, Guangzhou 510317, China; 3 University of South China’s Teaching Hospital, Guangdong Second Provincial General Hospital, Hengyang 421000, China

**Keywords:** preeclampsia, differentially expressed, dual-luciferase reporter gene, Western blot analysis

## Abstract

Despite its prevalence, preeclampsia (PE) remains unclear as to its etiology. Here, we aimed to investigate the mechanisms regulating differences in the gene expression of zinc-finger protein 516 (ZNF516) in the placenta. The expression of the placental ZNF516 gene and its association with critical clinical markers were verified, and a rigorous correlation analysis was conducted. With a dual-luciferase reporter gene assay, microRNA targeting the ZNF516 gene was predicted and confirmed. Finally, the molecular processes associated with ZNF516 were explored via microarray and bioinformatic analyses. In hypoxic conditions, miR-371-5p expression was reduced, resulting in ZNF516 expression being induced. Moreover, ZNF516 was shown to hinder trophoblast cell migration and invasion while enhancing trophoblast cell death in various *in vitro* cellular assays, such as cell counting kit-8, colony formation, wound healing, and Transwell assays. Our findings reveal a new regulatory network facilitated by ZNF516. ZNF516 overexpression inhibits trophoblast growth, movement, and penetration, potentially causing problems with placenta formation with the help of miR-371-5p suppression.

## INTRODUCTION

In pregnancy, preeclampsia (PE) is characterized by various degrees of placental malperfusion. [1]. PE causes 46,000 maternal deaths and over 500,000 fetal and infant deaths annually; thus, it is pertinent to address this issue [2, 3]. In early pregnancy, extravillous trophoblast cells must penetrate the decidua and undergo spiral artery transformation for placental development [4]. Insufficient extravillous trophoblast invasion into the uterine decidua and spiral artery remodeling can cause infertility and PE [5]. Infertility in women is a worldwide medical issue that several different abnormalities of the reproductive system, including PE, can bring on. However, limited progress has been achieved in effective clinical prediction, prevention, or treatment [6]. Because the clinical symptoms of PE are promptly alleviated after childbirth, PE is widely recognized as a placental disorder [7, 8]. PE process is commonly conceptualized as two distinct stages. One contributing factor is inadequate development of the uteroplacental spiral arteries and incomplete restructuring; the other involves disrupted blood flow between the uterus and placenta and increased oxidative stress inside the placenta. These factors collectively give rise to the observable symptoms of PE [9, 10]. Moreover, abnormal placental gene expression has been shown to result in trophoblast malfunction. Further clarification is still required to fully understand the regulatory involvement of these genes in the pathophysiology of PE [11].

MicroRNAs (miRNAs) are short RNA molecules comprising approximately 22 nucleotides. These noncoding RNAs are essential for regulating gene expression after transcription [12]. MiRNAs are generated by the placental trophoblast cells at the interface between the mother and fetus during pregnancy. The expression levels of these miRNAs change as gestational age progresses and placental development occurs. This observation underscores the crucial role of miRNAs in regulating placental functions [13]. A new study says that increasing miR-371-5p and decreasing PRPF4B speed up the G1/S shift in hepatocellular carcinoma cell lines, leading to more proliferating cells. MiR-371-5p expression has been linked to increased QGY-7703 cell proliferation *in vivo*, promoting the proliferation of a wide variety of tumor types.

However, the suppression of miR-371-5p results in contrasting outcomes [14]. It has been said that Hsa-miR-371-5p can help endometrial cancer start and spread. It is closely linked to endometrial growth [15]. A recent study revealed that zinc-finger protein 516 (ZNF516) (KIAA0222) is a Krüppel C2H2-type ZNF protein family member. Previous studies have provided evidence indicating that the protein ZNF516 substantially suppresses breast cancer cell growth and invasion in laboratory settings. This inhibition ultimately reduces breast cancer development and the metastasis of cancer cells to distant anatomical sites *in vivo* [16]. This study demonstrates a notable reduction in miR-371-5p levels within the placenta of individuals diagnosed with PE compared to those experiencing normal pregnant women. We then analyzed the direct binding of miR-371-5p to ZNF516, which leads to the inhibition of its expression. A series of cell-based studies were conducted to derive more conclusions.

## MATERIALS AND METHODS

### Placenta sample collection

In this study, placental tissue samples were collected from 89 patients diagnosed with PE and 71 healthy controls who underwent cesarean delivery at Guangdong No. 2 People’s Hospital between 2017 and 2019. Supplementary Table 1 summarizes the clinicopathological characteristics of the individuals with PE and healthy pregnant women. The hospital approved the protocol used in this study. All participants provided written consent after being fully informed about the study.

### RT-qPCR

RT-qPCR analysis was utilized to measure the levels of miR-371-5p and U6 using the Premix Ex Taq™ II kit (Takara, Japan) according to the manufacturer’s instructions, and the test was completed using the ABI 7500 PCR apparatus. To synthesize complementary DNA (cDNA), we extracted total RNA using TRIzol reagent (Carlsbad, CA, USA). Given the article’s length, we provide this section with the precise steps in the Supplementary Materials section of the article (Supplementary Table 2).

### Western blotting

The placental tissues were homogenized, and 100 μL of each tissue sample was placed in a reaction tube. The tissues were then lysed with 1 mL of cell lysate and protease for 30 minutes at 4°C with shaking once every 10 minutes. The lysed tissues were then centrifuged at 6000 rpm for 20 minutes to separate the components. The protein extract was collected after removing the lipid layer.

### Bioinformatics analysis

First, the GSE15789, GSE84260, and GSE96985 PE microarrays were acquired from the Gene Expression Omnibus database. Subsequently, a differential analysis was performed, with the criteria of |log FoldChange| >1 and a *P*-value < 0.05. The target genes of hsa-miR-371-5p were predicted using the TargetScan, miRDB, miRPathDB, and miRTargetLink databases. Additionally, the upregulated genes from the GSE96985 dataset were included in the analysis. The results of these predictions were visualized using a Venn diagram.

### Cell transfection

HTR-8/SVneo cells were genetically modified by introducing plasmids that expressed either (oe) ZNF516 or an inhibitor of miR-371-5p (anti-miR). All plasmids used in this experiment were obtained from GenePharma (China). The experimental procedure involved the utilization of Lipofectamine™ 2000 Transfection Reagent (11,668,019, Invitrogen, USA) according to the guidelines provided by the manufacturer. The measurement of expression levels was conducted 48 hours post-transfection utilizing either RT-qPCR or Western blotting techniques.

### Dual-luciferase reporter gene assay

Initially, the 3′-untranslated regions (3′-UTRs) of ZNF516’s wild-type (WT) and mutant (MUT) were synthesized and subsequently inserted into the pmiR-RB-REPORTTM vector (Guangzhou RiboBio Co., Ltd., China). Empty plasmid was the negative control. The WT and MUT plasmids were then cotransfected into HTR-8/SVneo cells with the miR-371-5p mimic, the corresponding mimic-NC, the miR-371-5p inhibitor, or the inhibitor NC. Afterward, the luminous signal was evaluated using dual-luciferase reporter analysis equipment manufactured by Promega (Madison, WI, USA).

### Cell viability assay

The assessment of cell viability was conducted with CCK-8 assays. The cells were preincubated at a temperature of 37°C with a CO_2_ concentration of 5% in a 96-well plate for a duration of 24 hours before cell transfection. After a period of 1, 2, 3, 4, and 5 days, HTR 8/SVneo cells were subjected to transfection with oe-ZNF516, anti-miR, or empty vectors. Subsequently, a volume of 10 μl of CCK-8 solution (Beyotime Biotechnology Co., Ltd., China) was introduced into each well. After that, the cellular culture was maintained for 4 hours, during which the optical density (OD) at a specific wavelength of 450 nm was measured using a microplate reader. GraphPad Prism 9.5 software was used to calculate and illustrate the OD values.

### Colony formation assay

Forty-eight hours after transfection, HTR8/SVneo cells were resuspended in serum-free medium for 24 hours. Then, 2 ml of the cells (approximately 800 cells) was plated in 60-millimeter dishes and cultivated for 14 days. The medium was replaced every 2 days. On the fourteenth day, the cells were subjected to a 10-minute treatment with 4% polyformaldehyde, followed by a 10-minute staining with 0.1% crystal violet. More than 50 cell colonies per dish were counted using an Olympus microscope.

### Wound healing assay

The evaluation of cellular migration was conducted utilizing a wound healing assay. HTR48/SVneo cells transfected with oe-ZNF516, anti-miR, or empty vectors were seeded in six-well plates at 48 hours posttransfection. Scratched regions were imaged using a microscope (EVOS FL Automated Imaging System, Life Technologies, Carlsbad, CA, USA) at 0, 24, and 48 hours, and wound healing rates were quantified using ImageJ software.

### Transwell assay

An 8-μm Transwell filter from Corning Incorporated (Corning, NY, USA) was used to test the invasion potential of HTR-8/SVneo cells. Matrix gel (50 μl) from BD Biosciences (La Jolla, CA, USA) was applied to the apical chamber of the basement membrane. After transfection with oe-ZNF516, anti-miR, or empty vectors, HTR8/SVneo cells were introduced into the apical chamber using serum-free media. The media in the basolateral chamber were supplemented with 20% fetal bovine serum (FBS). The cells were grown for 48 hours with 5% CO_2_ at 37°C to assess their invasive ability.

### Statistical analysis

GraphPad Prism version 9.5 (San Diego, CA, USA) and IBM SPSS Statistics for Windows, version 29.0, were used for the statistical analysis. The clinical samples were subjected to triple analysis to mitigate the potential influence of systematic and random errors/bias. Student’s *t*-test was used to compare two groups. The relationship between two components was assessed using linear regression analysis. The data were analyzed using Pearson correlation analysis to determine the correlations between the variables. The data were subjected to hierarchical clustering analysis using Origin 2024 software (OriginLab, Northampton, MA, USA). An analysis was conducted to examine the relationship between clinical characteristics and various components of the principal component analysis (PCA) data.

### Data availability

The data employed in this article will be made available to the corresponding author upon a reasonable request.

## RESULTS

### Reduced miR-371-5p is involved in PE progression

**Figure 1:**
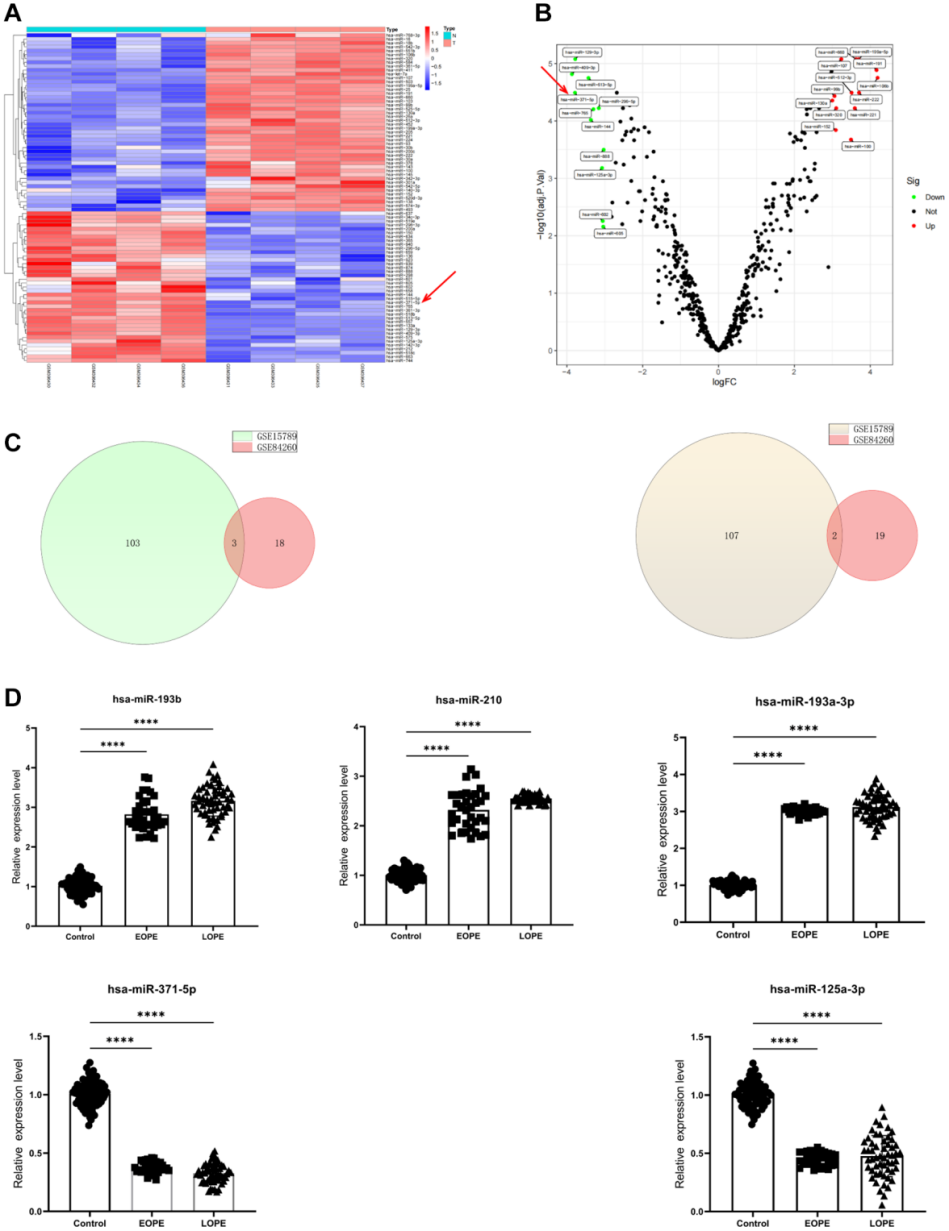
**MiR-371-5p expression is decreased in patients with preeclampsia.** (**A**, **B**) Volcano plot and heatmap showing DEGs in GSE15789 (*n* = 215, *P* < 0.05, red arrows, miR-371-5p). (**C**) The GSE15789 and GSE84260 datasets’ upregulated (left panel) and downregulated genes (right panel) are shown in Venn diagrams. (**D**) The present study involved the utilization of RT-PCR in analyzing the expression levels of hsa-miR-193b, hsa-miR-210, hsa-miR-193a-3p, hsa-miR-371-5p, and hsa-miR-125a-3p in the placenta. Control, *n* = 71; EOPE, *n* = 34; and LOPE, *n* = 55. ^****^P < 0.0001.

The microarray data GSE15789 demonstrated that 106 miRNAs exhibited a considerable increase in expression, while 109 miRNAs displayed a significant decrease in expression among individuals diagnosed with PE (Figure 1A, 1B). A comprehensive set of stringent screening criteria was used to identify 21 downregulated miRNAs and 21 upregulated miRNAs from the GSE84260 dataset. These miRNAs were then represented in a Venn diagram (Figure 1C). Three gene intersections exhibited upregulated expression, namely, hsa-miR-193b, hsa-miR-210-3p, and hsa-miR-193a-3p. Conversely, two gene intersections, specifically hsa-miR-371-5p and hsa-miR-125a-3p, were downregulated.

Placental specimens were collected from 89 individuals diagnosed with PE and 71 with normotensive blood pressure. Supplementary Table 1 displays the individuals’ clinical features. The research findings indicated significant discrepancies in many health indicators, including blood pressure, albuminuria, body mass index, liver and kidney function markers, and birth weight, between the two groups. The RT-qPCR test showed that the levels of hsa-miR-193b, hsa-miR-210, and hsa-miR-193a-3p were significantly higher in the placentas of 34 people with early-onset PE (EOPE) and 55 people with late-onset PE (LOPE). Hsa-miR-371-5p and hsa-miR-125a-3p levels were also lower in the placentas that were being studied (Figure 1D).

### ZNF516 expression is upregulated in the placenta of PE patients

**Figure 2:**
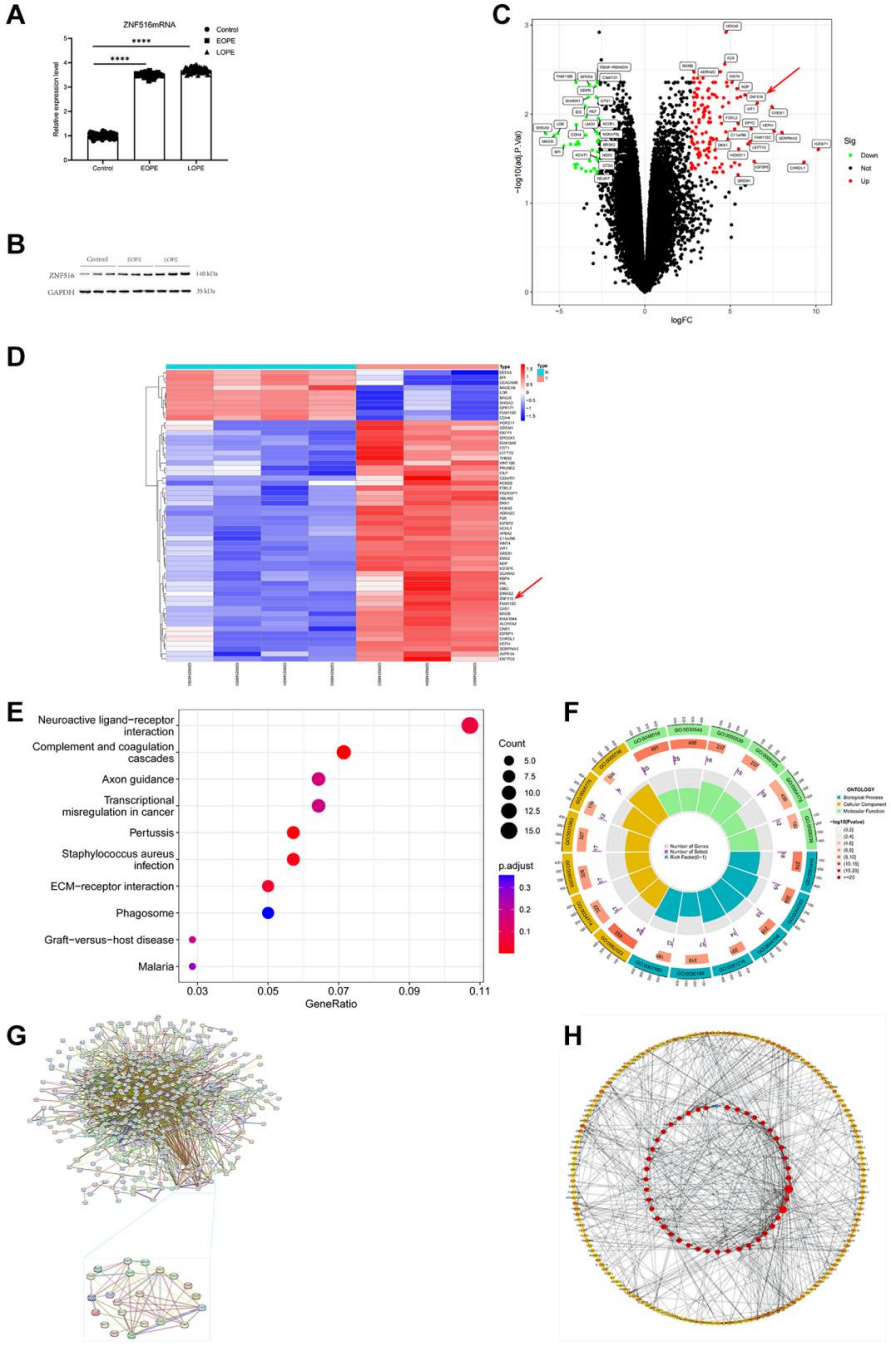
**ZNF516 expression in the placentas of individuals with PE and those with pregnancies without abnormalities.** (**A**) This study employed the RT-qPCR technique to evaluate the mRNA expression levels of ZNF516 in the placenta of both the EOPE and LOPE groups. Control, *n* = 71; EOPE, *n* = 34; and LOPE, *n* = 55. ^****^P < 0.0001. (**B**) Western blotting was used to assess the protein expression of ZNF516 in placental tissue. *n* = 3. (**C**, **D**) Volcano plot and heatmap showing the DEGs in the GSE96985 dataset. *n* = 308, *P* < 0.05, red arrows, ZNF516. (**E**) KEGG pathway analysis of the biological processes associated with the upregulated DEGs. (**F**) The DEGs primarily exhibit enhanced GO keywords related to biological processes, cellular components, and molecular functions. (**G**, **H**) Through the utilization of cytoHubba, the PPI network was utilized to choose the top 30 hub genes.

The study involved the collection of 89 placental samples from women diagnosed with PE, while 71 samples were taken from women with normal blood pressure. The RT-qPCR analysis results indicated that considerable upregulation of ZNF516 mRNA expression in placentas affected EOPE and LOPE (Figure 2A). Subsequently, Western blotting analysis confirmed an increase in the protein level of ZNF516 (Figure 2B). A total of 203 upregulated mRNAs were identified from the GSE96985 database. Conversely, 105 mRNAs were downregulated (Figure 2C, 2D). The present study confirmed that there is an upregulation of ZNF516 expression in patients who have been diagnosed with PE. To further investigate the potential impact of ZNF516 on the molecular biology of cells, an analysis was conducted first utilizing the Gene Ontology (GO) and then the Kyoto Encyclopedia of Genes and Genomes (KEGG) GSE96985 databases (Figure 2E, 2F). The analyses revealed disparities in genetic pathways, suggesting the participation of these genes in several biological processes, including connective tissue creation, cellular growth, and epithelial cell proliferation. Analysis of protein-protein interactions (PPI) validated the network and identified significant differentially expressed genes (DEGs). Among the six essential genes, ZNF516 (degree = 8), PRDM16 (degree = 12), RCOR1 (degree = 12), and CDH1 (degree = 61) were upregulated, and BICC1 (degree = 5) and KDM1A (degree = 16) were downregulated (Figure 2G, 2H).

### ZNF516 is a direct downstream target of miR-371-5p

**Figure 3:**
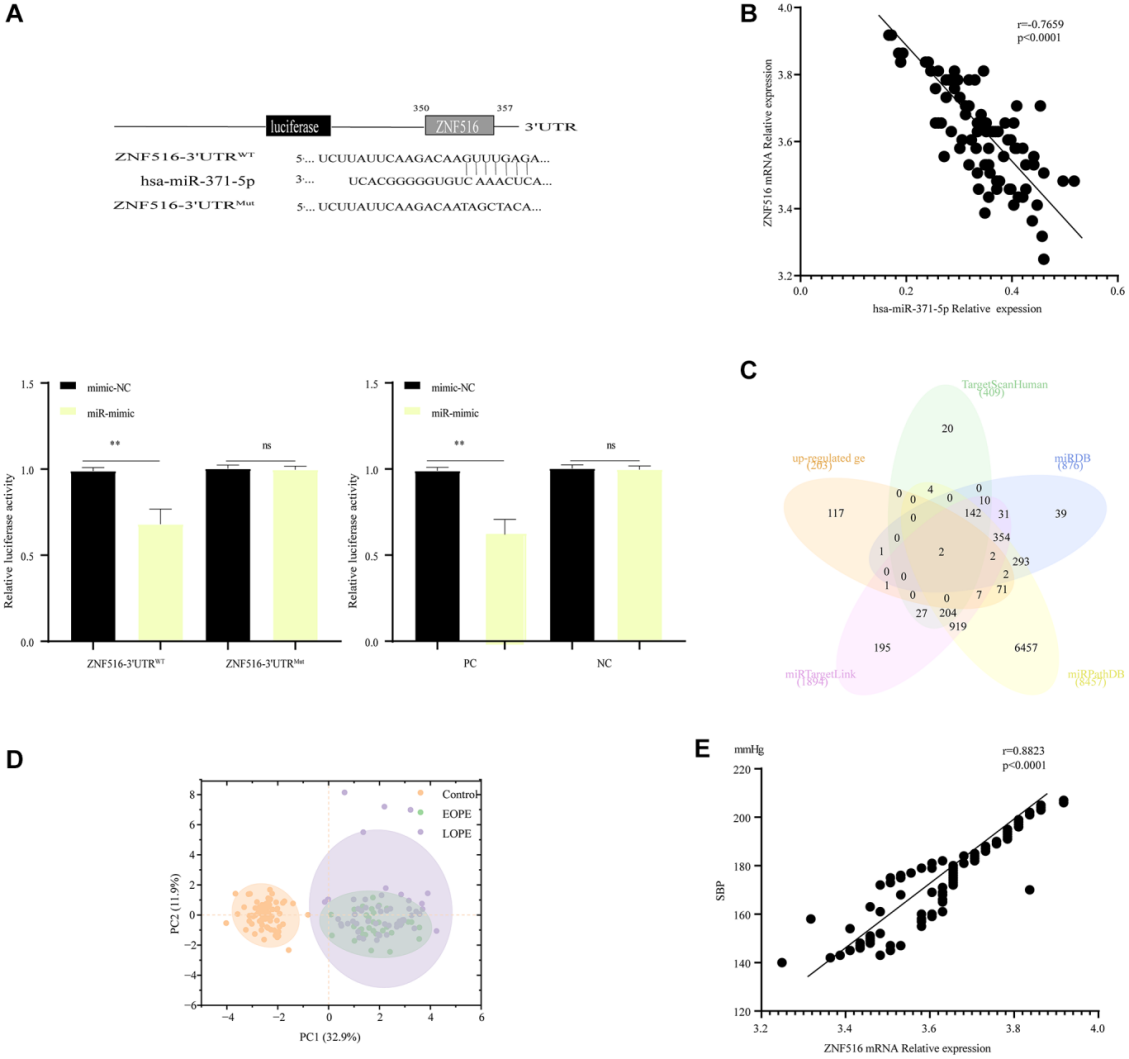
**MiR-371-5p suppressed trophoblast cell proliferation, migration, and invasion via lowering ZNF516 expression.** (**A**) The sequences of hsa-miR-371-5p and the 3′ untranslated region (3′UTR) of ZNF516, where it is believed to bind. The upper panel illustrates the specific nucleotides altered in the ZNF516-3′UTR mutant. The luciferase activity of ZNF516 with a wild-type 3′-UTR was dramatically inhibited by miR-371-5p; however, no effect on ZNF516 with a mutant 3′-UTR was observed. Conversely, the luciferase activity of ZNF516 cells increased after treatment with anti-miR-371-5p, as shown in the lower panel. The statistical significance levels are ^*^P < 0.05 and ^**^P < 0.01. In this context, NC refers to the negative control, representing the wild-type mutant. (**B**) The Venn diagram displays target genes of hsa-miR-371-5p predicted by the TargetScan, miRDB, miRPathDB, and miRTargetLink websites and the upregulated genes in the GSE96985 dataset. (**C**) A scatter map was generated to illustrate the significant inverse association between the levels of hsa-miR-371-5p and ZNF516 mRNA in 89 placental samples from patients with PE. (**D**) After incorporating batch effects as covariates into the linear model, principal component analysis (PCA) plots are provided for each of the 160 samples. The plot displays individual samples, which are represented by dots. The dots are colored according to the disease condition: yellow for healthy pregnant women (control), purple for patients with early-onset preeclampsia (EOPE), and green for patients with late-onset preeclampsia (LOPE). (**E**) A scatter plot was generated to illustrate the significant inverse association observed between the levels of ZNF516 mRNA and SBP in a sample of individuals with PE. *n* = 89, *P* < 0.0001. Abbreviation: SBP: systolic blood pressure.

The sequence of hsa-miR-371-5p was identified in the 3′-UTR (untranslated region) of ZNF516 mRNA through the use of TargetScan (Supplementary Table 3). It was shown that miR-371-5p significantly inhibited the activity of the luciferase enzyme of ZNF516 exclusively in the presence of the wild-type ZNF516 3′UTR (Figure 3A). In addition, hsa-miR-371-5p target genes were identified using the TargetScan miRDB, miRPathDB, and miRTargetLink databases, and the GSE96985 database revealed 203 mRNAs in the intersection. Intersecting genes ZNF516 and ITGB8 were identified (Figure 3B). It was further verified that ZNF516 is a target gene of miR-371-5p. hsa-miR-371-5p was linked with ZNF516 (r = −0. 7659, *P* < 0. 001; Figure 3C). Principal Component Analysis (PCA) also revealed a distinction between the control group and the EOPE and LOPE groups, as depicted in Figure 3D. The clinical values of 17 parameters (Supplementary Table 1) were subjected to the Kaiser-Meyer-Olkin test (KMO) and Bartlett’s test of sphericity statistics using SPSS software. The purpose of this analysis was to assess the grouping characteristics of the 160 samples and examine the relationships between clinical attributes. Bartlett’s test of sphericity yielded X^2^ = 1341.87 (*P* < 0.001), while the KMO index was 0.787. ZNF516 exhibited a positive linear correlation with SBP (r = 0. 8823, *P* < 0. 0001; Figure 3E).

### ZNF516 inhibits the viability, proliferation, and invasion of trophoblast cells *in vitro*

**Figure 4:**
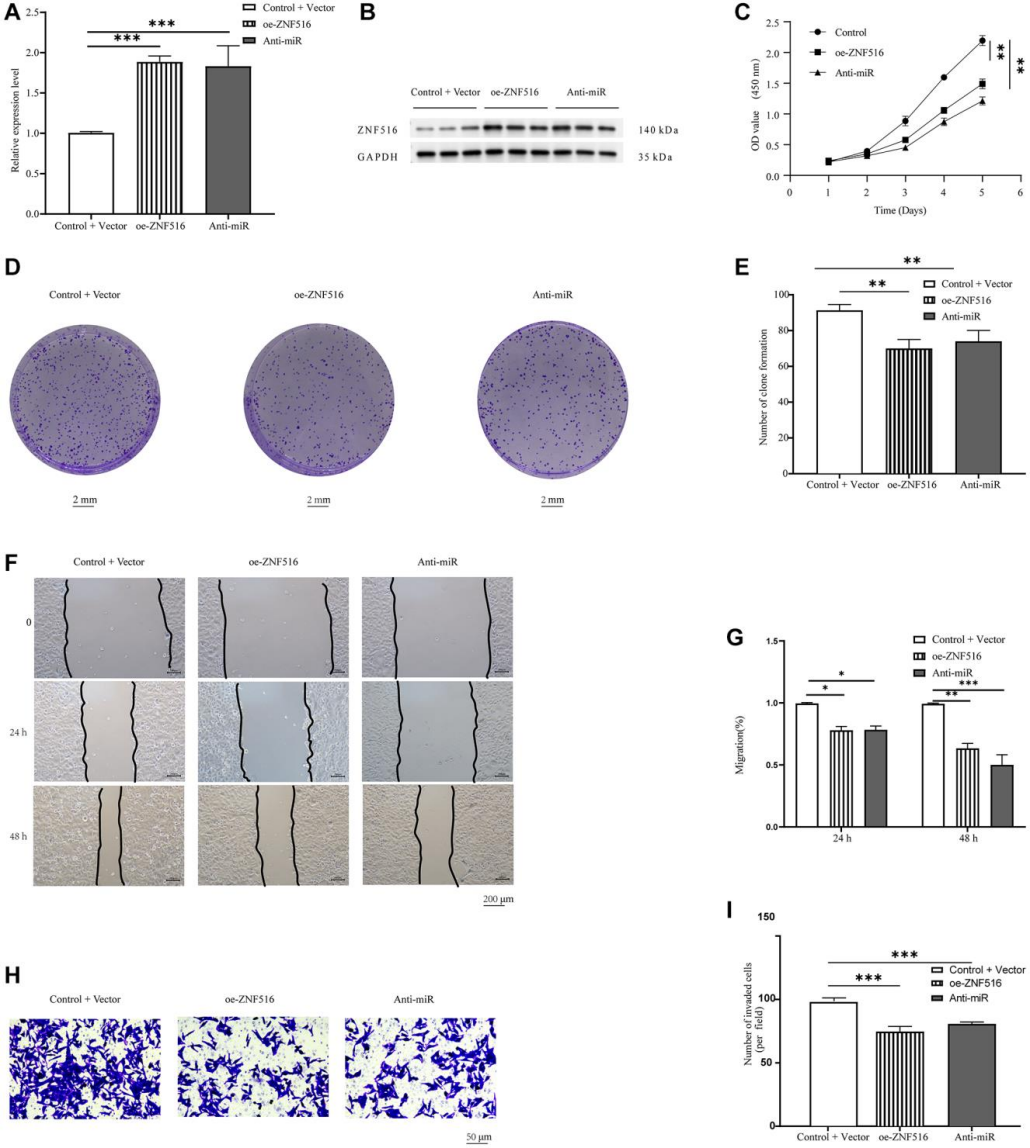
***In vitro*, ZNF516 inhibits trophoblast migration, invasion, and viability.** (**A**) RT-qPCR was used to measure how much ZNF516 was expressed in HTR8/SVneo cells after they were treated with oe-ZNF516 and anti-miR. (**B**) Western blotting was used to measure the level of ZNF516 in HTR-8/SVneo cells that had been treated with oe-ZNF516 and anti-miR-371-5p. (**C**) ZNF516 reduced the viability of HTR48/SVneo cells. The vitality of HTR 8/SVneo cells was assessed using CCK-8 assays after transfecting them with oe-ZNF516, anti-miR, or empty vectors for durations of 1, 2, 3, 4, or 5 days. (**D**) The proliferation potential of HTR8/SVneo cells was evaluated through the use of a colony formation test. The growth of HTR8/SVneo cell colonies was reduced by ZNF516 (scale bar = 2 mm). (**E**) Colonies were quantified using ImageJ software. (**F**, **G**) We used wound healing experiments to look at how overexpressing ZNF516 affected the ability of HTR48/SVneo cells to move. HTR48/SVneo cells transfected with oe-ZNF516, anti-miR, or empty vectors were seeded in six-well plates 48 hours posttransfection. Images were captured at 0, 24, and 48 hours. Scratched wound boundaries are marked by black lines (scale bar = 200 μm). (**H**, **I**) HTR8/SVneo cell migration and invasion were measured using Transwell assays (scale bar = 50 μm). *N* = 3; all results are presented as means ± SEMs. ^*^P < 0.05, ^**^P < 0.01, and ^***^P < 0.001, respectively, in comparison to the vector + control group. ZNF516, overexpressed-ZNF516; anti-miR, miR-371-5p inhibitors.

After conducting additional research, we investigated the molecular mechanism that ZNF516 uses to control the behavior of trophoblast cells. Initially, we used RT-qPCR and Western blotting to look at ZNF516 expression in HTR-8/SVneo cells treated with either oe-ZNF516 or anti-miR. There was a significant increase in ZNF516 expression in trophoblasts in the oe-ZNF516 and anti-miR groups compared to the control + vector group (*P* < 0.001, Figure 4A, 4B). Then, we examined the proliferation of trophoblasts transfected with either oe-ZNF516 or anti-miR using CCK-8 assays. ZNF516 overexpression reduced the trophoblast proliferation rate (*P* < 0.01, Figure 4C). The results of the colony formation experiments demonstrated a significant inhibition of trophoblast colony formation upon overexpression of ZNF516 (*P* < 0.01, Figure 4D, 4E). Furthermore, it was observed by wound healing assays that the migration capacity of HTR 8/SVneo cells was diminished upon overexpression of ZNF516 (*P* < 0.05, Figure 4F, 4G); this finding is consistent with the findings of the Transwell assays, which also revealed that ZNF516 overexpression significantly slowed trophoblast invasion (*P* < 0.001, Figure 4H, 4I). Collectively, our findings indicate that ZNF516 hinders the viability, proliferating, and invasion of HTR 8/SVneo cells.

## DISCUSSION

PE is widely acknowledged, but its pathophysiology remains incompletely comprehended. Insufficient invasion of trophoblasts and aberrant adaptive placentation are widely accepted to be the underlying pathogenic mechanisms of PE [17]. This study presented empirical findings indicating increased ZNF516 levels among women diagnosed with PE. ZNF516 upregulation resulting from miR-371-5p decrease suppressed the biological activity of trophoblast cells. Numerous studies have documented the presence of ZNF domains in a substantial proportion (approximately 5%) of human proteins. Initially identified for their ability to attach to DNA, ZNF proteins exhibit remarkable versatility in their binding capabilities. In addition to binding to DNA, ZNFs have been found to bind to several other molecules, including RNA, lipids, proteins, and posttranslationally modified proteins [18]. The transcriptional coactivator CBP is the sole protein with a transcriptional adaptor zinc (TAZ) domain. In CBP, two ZNFs combine to produce a distinct fold that is not present in other known ZNFs. In conclusion, CBP functions as an acetyltransferase and is attracted to DNA through interactions with various DNA-binding transcription factors. Nevertheless, the TAZ domains are crucial in facilitating many of these interactions. For example, the N-terminal TAZ1 domain exhibits binding affinity for hypoxia-inducible element 1a (HIF-1a). Specifically, HIF-1a recruits CBP when oxygen availability is limited, facilitating the regulation of gene expression required for cellular survival [19]. Pertinent scholarly investigations have established that the deacetylase HDAC6 possesses consecutive catalytic domains and a ubiquitin-binding ZNF. The catalytic domain demonstrates antiviral properties; however, ZNF promotes influenza A virus infection and triggers cellular stress responses, which is unexpected [20]. Accumulating evidence shows that miRNAs are crucial to controlling approximately 60% of protein-coding genes. Furthermore, miRNAs have been found to influence several cellular processes, such as the epithelial–mesenchymal transition, which facilitates the cellular acquisition of migratory and invasive capabilities [21, 22]. Researchers have shown that miRNAs regulate trophoblast biological processes and influence the onset and progression of PE [23, 24]. MiRNAs are believed to possess specificities regarding spatial distribution, temporal regulation, and tissue/cellular localization. These particular characteristics lead to their participation in the formation of tissues, the occurrence of developmental events, and the processes of cellular differentiation [25]. A study has demonstrated that miRNA-371-5p indicates a prevailing expression level in both cytotrophoblasts and syncytiotrophoblasts within the placenta throughout the initial trimester. In addition, miRNA-371-5p levels increase significantly during the last few weeks of the first trimester in syncytiotrophoblasts, villous stromal cells, and fetal arteries [26]. Furthermore, the expression of miR-371-5p has been observed to be increased in gastric cancer. MiR-371-5p is a giant tumor suppressor homolog two regulator that facilitates testicular cancer spread and growth [27].

In summary, this investigation provides evidence that ZNF516 acts as a mediator connecting hypoxia with PE development. Our research confirms that hypoxia induces HIF-1a by controlling miR-371-5p, which affects the expression of ZNF516. Using miR-371-5p as a biomarker for PE in future studies, we confirmed that ZNF516 regulates cell proliferation, migration, and invasion in trophoblast cells. Researchers previously focused solely on the diagnostic effectiveness of single markers, neglecting the importance of comprehensive analyses. These findings may illuminate PE subtype processes. In addition, forthcoming *in vivo* investigations will elucidate the scientific importance of miR-371-5p and ZNF516 as molecular markers, and further clinical application will contribute to better allocation of clinical resources, preventing unnecessary hospital admissions and surgeries in low-risk patients and deepening our understanding of the roles of these markers in PE pathogenesis.

## Supplementary Materials

Supplementary Methods

Supplementary Tables
