# Supplementary material for: MiR-371-5p regulates trophoblast cell proliferation, migration, and invasion by directly targeting ZNF516
Source: Aging (Albany NY). 2024 May 17;16(10):8585–98. doi: 10.18632/aging.205826 (PMC11164490; doi:10.18632/aging.205826)
Supplement: Supplementary Methods [file aging-16-205826-s001.pdf]

Supplementary Materials

RT-qPCR

RT-qPCR analysis was utilized to measure the levels of miR-371-5p and U6 using a TaqMan reverse transcription kit and a TaqMan miRNA assay kit according to the manufacturer's instructions. A total of 1 µg of RNA was reverse-transcribed into cDNA. RT-qPCR was performed with the following thermocycling conditions: initial denaturation at 95°C for 5 min, followed by 40 cycles of denaturation at 95°C for 15 sec, and annealing at 60°C for 30 sec. The products were identified using melting curve analysis. The $2^{-\Delta\Delta Ct}$ method was used to assess the relative expression levels, with U6 and GAPDH used as internal reference genes. The trials were performed in triplicate, and the primers used are listed in Supplementary Table 2.

Western blotting

The placental tissue was cleaved in cleavage buffer (Biosharp, Hefei, Anhui, China) supplemented with a protease inhibitor cocktail (MedChemExpress, Monmouth, NJ, USA). The protein concentration was measured using a Pierce BCA protein detection kit (reference: 23225, Thermo Fisher Scientific, Waltham, MA, USA). The protein lysate (50 µg) was loaded onto a 10% SDS-PAGE gel and then transferred to a PVDF membrane (Millipore, Billerica, MA, USA). The membrane was then incubated with 5% skim milk at room temperature for 1 hour, followed by incubation with primary antibodies against GAPDH (ab8245) and rabbit anti-ZNF516 (ab121486) at dilutions of 1:2000 and 1:2000, respectively, for Western blotting. The incubation procedure was conducted overnight at 4°C. The membrane was washed three times for 10 minutes each and then incubated at room temperature with horseradish peroxidase bound to a secondary antibody (1-1000, 7074 S, Cell Signaling Technology, Danvers, MA, USA) for 2 hours. The membrane was subsequently washed three times for 10 minutes each, developed using an enhanced chemiluminescence reagent (Biomiga, San Diego, CA, USA), and observed with an X-ray device.
