# Supplementary material for: MiR-371-5p regulates trophoblast cell proliferation, migration, and invasion by directly targeting ZNF516
Source: Aging (Albany NY). 2024 May 17;16(10):8585–98. doi: 10.18632/aging.205826 (PMC11164490; doi:10.18632/aging.205826)
Supplement: Supplementary Tables [file aging-16-205826-s002.pdf]

SUPPLEMENTARY TABLES

Supplementary Table 1. The clinical characteristics of the participants (Mean ± SD).

	Control *n* = 71	EOPE *n* = 34	LOPE *n* = 55
Age (years)	31. 15 ± 4. 99	31. 71 ± 4. 52	30. 02 ± 4. 35
Gestational Weeks	37. 82 ± 1. 35	31. 68 ± 0. 85	42. 67 ± 18. 71
Hb (g/L)	117. 61 ± 17. 50	112. 40 ± 18. 12	104. 5 ± 26. 42
BMI (kg/m2)	24. 47 ± 4. 21	26. 73 ± 4. 08	26. 61 ± 4. 72
D-DIC (mg/L)	0. 54 ± 0. 19	1. 97 ± 0. 82	1. 95 ± 0. 78
FIB (g/L)	4. 34 ± 1. 01	5. 20 ± 1. 64	5. 32 ± 1. 45
Neonatal Weight (kg)	3. 16 ± 0. 65	1. 35 ± 0. 37	2. 90 ± 0. 63
24UPro (mg/24 h)	133. 71 ± 73. 74	1006 ± 556. 4	870. 5 ± 598. 2
Scr (umol/L)	50. 96 ± 12. 21	55. 24 ± 17. 67	60. 67 ± 18. 35
PLT (10^9/L)	185. 30 ± 50. 75	175. 60 ± 60. 72	153. 70 ± 71. 04
ALT (U/L)	16. 14 ± 6. 93	51. 62 ± 14. 64	53. 09 ± 14. 92
AST (U/L)	22. 07 ± 7. 05	40. 82 ± 13. 02	42. 58 ± 13. 56
SBP (mmHg)	109. 80 ± 10. 89	163. 80 ± 14. 88	178. 60 ± 17. 28
DBP (mmHg)	66. 79 ± 7. 83	83. 00 ± 9. 58	85. 96 ± 17. 08
WBC ($^*10^9$/L)	9. 38 ± 2. 49	11. 28 ± 1. 94	12. 08 ± 2. 52
PMN($^*10^9$/L)	5. 85 ± 1. 59	8. 46 ± 1. 62	9. 04 ± 2. 05
Total Ca2+(mmol/L)	2. 35 ± 0. 21	2. 03 ± 0. 089	2. 05 ± 0. 12

Supplementary Table 2. The primer sequences for PCR.

Gene name	Primer orientation	Sequences
	Forward	5′-GGACCCGGTCAACAGCTAC-3′
ZNF516	Reverse	5′-GGCCCCGTACTTGAGGTACT-3′
	Forward	5′-GGAGCGAGATCCCTCCAAAAT-3′
GAPDH	Reverse	5′-GGCTGTTGTCATACTTCTCATGG-3′
	Forward	5′-ACACTCCAGCTGGGAACTGGCCCTCAAAGT-3′
hsa-miR-193b	Reverse	5′-CTCAACTGGTGTCGTGGAGTCGGCAATTCAGTTGAG AGCGGGAC-3′
	Forward	5′-ACACTCCAGCTGGGCTGTGCGTGTGACAGC-3′
hsa-miR-210-3p	Reverse	5′-CTCAACTGGTGTCGTGGAGTCGGCAATTCAGTTGAGTCAGCCGC-3′
	Forward	5′-ACACTCCAGCTGGGAACTGGCCTACAAAGT-3′
hsa-miR-193a-3p	Reverse	5′-CTCAACTGGTGTCGTGGAGTCGGCAATTCAGTTGAGACTGGGAC-3′
	Forward	5′- ACACTCCAGCTGGGACTCAAACTGTGGG-3′
hsa-miR-371-5p	Reverse	5′-CTCAACTGGTGTCGTGGAGTCGGCAATTCAGTTGAG AGTGCCCC-3′
	Forward	5′-ACACTCCAGCTGGGACAGGTGAGGTTCTTG -3′
hsa-miR-125a-3p	Reverse	5′-CTCAACTGGTGTCGTGGAGTCGGCAATTCAGTTGAGGGCTCCCA-3′
	Forward	5′-CTCGCTTCGGCAGCACA-3′
U6snRNA	Reverse	5′-AACGCTTCACGAATTTGCGT-3′

Supplementary Table 3. The binding site of ZNF516 to hsa-miR-371-5p through the use of TargetScan (https://www.targetscan.org/vert_80/).

Conserved								
	**Predicted consequential pairing of target region (top) and miRNA(bottom)**	**Site type**	**Context ++ score**	**Context++ score percentile**	**Weighted Context++ score**	**Conserved branch length**	**PCT**	**Predicted relative Kp**
Position 350-357 of ZNF5163′UTR hsa-miR-371a-5p	5′...UCUUAUUCAAGACAAGUUUGAGA.. 3′...UCACGGGGGUGUCAAACUCA..	8mer	−0.03	82	−0.03	2.049	N/A	−4.864
